# Laboratory-based surveillance in Latin America: attributes and limitations in evaluation of pneumococcal vaccine impact

**DOI:** 10.1080/21645515.2021.1972709

**Published:** 2021-10-07

**Authors:** Javier Nieto Guevara, Adriana Guzman-Holst

**Affiliations:** Emerging Markets, GSK Vaccines, Panama City, Panama

**Keywords:** Argentina, Brazil, Chile, Colombia, Ecuador, Mexico, pneumococcal conjugate vaccines, invasive pneumococcal disease, children, surveillance

## Abstract

Disease surveillance data are needed to monitor trends in disease activity, inform decision-making in public health and evaluate disease prevention/control measures. The Sistema Regional de Vacunas (SIREVA) supports laboratory-based surveillance of invasive pneumococcal disease (IPD) in Latin American countries, providing information on identification, distribution, and anti-microbial susceptibility of pneumococcal strains. We estimated the proportion of pneumococcal meningitis and sepsis/bacteremia cases captured by SIREVA, by comparing the number of SIREVA-reported isolates in Argentina, Brazil, Chile, Colombia, Ecuador and Mexico with the estimated expected number of cases based on regional estimates of disease incidence. In all six countries, the number of isolates reported by SIREVA was consistently lower than the number of cases expected, across all years with data available. The proportion of SIREVA-reported isolates was highest in Chile (43–83%) and lowest in Mexico (1.4–3.5%). Passive surveillance systems such as SIREVA are important tools for monitoring circulating strains that could be related to pneumococcal disease, but our results show that SIREVA is likely to underestimate pneumococcal disease incidence. This under-reporting will limit the precision of surveillance data in monitoring changes in the incidence of IPD after vaccine introduction, and this should be considered when assessing the impact of vaccination programs.

## PLAIN LANGUAGE SUMMARY

What is the context?

- Infectious disease surveillance is an important epidemiological tool to monitor the health of a population.
- Surveillence can be used to detect trends in disease activity and to trigger disease control measures.
- In Latin America, the SIREVA surveillance system monitors occurrence of bacterial pneumonia, sepsis/bacteremia and meningitis.
- However, passive surveillence systems may understimate disease occurrence.

What is new?

- We compared the number of isolates of invasive pneumococcal disease (IPD), specifically meningitis and sepsis/bacteremia, in children aged <5 years reported in SIREVA data in six countries in Latin America with the expected number of cases based on regional estimates of IPD incidence.
- Our results show that the number of isolates reported by SIREVA was consistently lower than the estimated number of cases, across all six countries and all the years available.
- The percent difference between SIREVA-reported isolates and estimated number of cases was variable between countries, ranging from 43-83% in chile to 1.4-3.5% in Mexico.

What is the impact?

- Passive surveillance systems such as SIREVA are important tools for monitoring disease incidence, but they are likely to underestimate pneumococcal disease occrruence.
- This under-reporting will limit the precision of surveillance data in monitoring changes in disease incidence after vaccine introduction, and this needs to be considered when assessing vaccine impact.

## Introduction

Infectious disease surveillance is an important epidemiological tool to monitor the health of a population. Surveillance data are needed to describe the current burden and epidemiology of disease and to monitor trends and patterns in disease activity.^1^ They can be used to detect unusual disease patterns and trigger disease control measures, to inform resource allocation decisions in public health, and to evaluate disease control and prevention initiatives.^2^ In passive surveillance systems, medical professionals in the community and at health facilities report cases to the public health authorities, which conduct data management and analysis once the data are received. Public health staff do not engage in identifying cases but rather assess data completeness and reliability of the reported cases. In contrast, active surveillance requires public health staff to engage actively in the system and take action in order to receive reports of disease cases.^1^

The Pan-American Health Organization (PAHO) recommends that countries should conduct surveillance of pneumonias and meningitis in children aged <5 years to assess disease burden and population profile.^3,4^ The Sistema Regional de Vacunas or Regional Vaccine System (SIREVA) is a network of laboratories conducting surveillance of bacterial pneumonia, sepsis/bacteremia and meningitis in Latin America since 1993, organized by PAHO. The surveillance program identifies the main bacterial pathogens responsible for pneumonia, sepsis/bacteremia and meningitis, identifies circulating serotypes and characterizes their susceptibility to common antibiotics.^4^ The SIREVA II project (Network Surveillance System for the Bacterial Agents Responsible for Pneumonia and Meningitis) was launched by PAHO in 2005 to strengthen regional surveillance of disease following the availability of pneumococcal conjugate vaccines (PCV) in the region. Currently, this multicenter, multicountry, passive laboratory surveillance network includes 19 national reference laboratories in 19 countries.^5,6^ However, SIREVA was not designed to assess disease burden, changes in disease incidence, or vaccine impact because this surveillance does not have population denominators.

Surveillance information can be used to support decision-making on vaccine introduction, to assess circulating strains and potential usefulness of vaccines, and to provide guidelines for antibiotic use.^4^ Passive laboratory surveillance allows monitoring the prevalence of pneumococcal serotypes causing disease in children.^5^ However, PAHO recommends that countries also implement active and/or sentinel surveillance before and after vaccine introduction.^7^ Approximately 11 of the 32 Latin American countries that have introduced PCVs in their national immunization programs began surveillance before vaccine introduction.^7^ However, surveillance alone is insufficient to determine disease incidence, and confounding factors such as quality of surveillance between years prevent definitive conclusions.^7^ The assessment of PCV impact based on national passive surveillance systems is challenging due to timely and limited data availability, and heterogeneity in approach to case reporting and laboratory diagnosis of cases.^5,7^ Since reporting to laboratory-based systems such as SIREVA is not compulsory in Latin American and Caribbean countries (except for pneumococcal meningitis), passive surveillance is likely to underestimate disease occurrence.^5^

**Figure 1.**
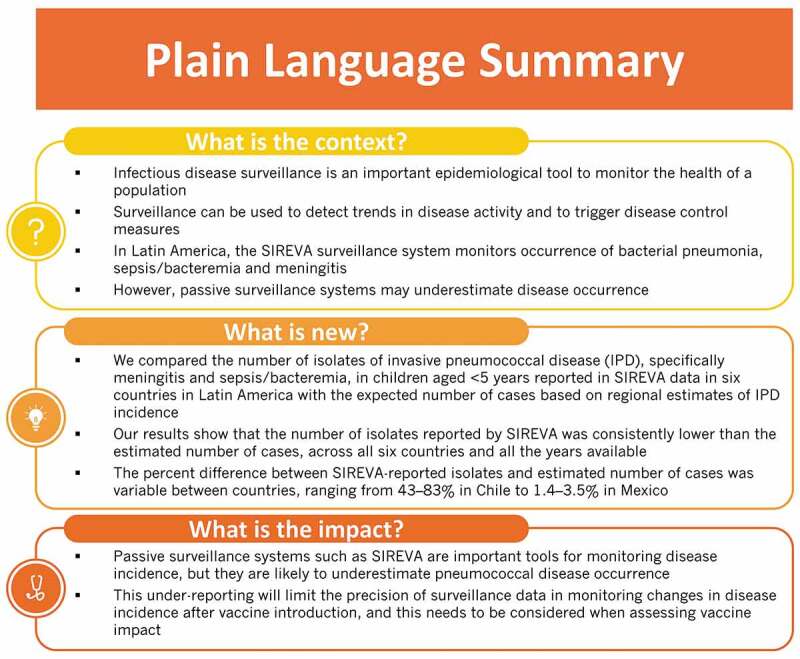
Plain language summary.

The purpose of this analysis was to compare the number of isolates of invasive pneumococcal disease (IPD), specifically meningitis and sepsis/bacteremia, in children aged < 5 years reported in SIREVA data in six countries with the expected number of cases based on regional estimates of IPD incidence. The goal of SIREVA was never to capture all cases. This analysis will enable us to estimate the proportion of cases captured by the surveillance program. Figure 1 provides a plain language summary.

## Methods: isolates reported in SIREVA surveillance data

For six countries in Latin America (Argentina, Brazil, Chile, Colombia, Ecuador and Mexico), we extracted annual data from the SIREVA reports on the number of pneumococcal meningitis and sepsis/bacteremia isolates in children aged < 5 years, from the year of PCV introduction to the latest year for which SIREVA data are available.

The year in which higher-valent PCV was introduced into the national vaccination program in each country was considered as a transition year. In Argentina^8^ and Colombia^9^ this transition year was 2012, in Brazil it was 2010,^10^ and in Chile,^11^ Ecuador^12^ and Mexico^13^ it was 2011. The latest year for which locally-published SIREVA data were available was 2019 for Argentina, Brazil and Mexico. In Chile, Colombia and Ecuador, no clinical diagnosis data were available from 2018, so the latest year with regionally-published SIREVA data available for this analysis was 2017.

## Methods: estimating the expected number of cases

Estimates of the incidence of pneumococcal meningitis and sepsis/bacteremia cases worldwide in children aged <5 years before PCV introduction were published in 2009.^14^ In the Americas region, the incidence of pneumococcal meningitis was estimated at 12 per 100,000 (range 9 to 17), and the incidence of non-pneumonia-non-meningitis IPD, referred to here as sepsis/bacteremia, was estimated at 15 per 100,000 (range 11 to 21).^14^ We estimated the total expected number of pneumococcal meningitis and sepsis/bacteremia cases in the same six countries in Latin America for which we obtained SIREVA data, by multiplying this Americas region incidence rate by the estimated annual <5 year-old population of each country obtained from United Nations estimates,^15^ using the following formula:

$$Expected\;number\;of\;cases=\frac{\begin{array}{c} IPD\;incidence\;rate*\\ Annual\;population \end{array}}{100,000}$$

We estimated expected annual case numbers from the transition year to the year of the most recent available SIREVA surveillance data in each country.

The study published by O’Brien et al^14^ was conducted before PCV introduction in Latin America. We therefore needed to adjust the expected case numbers to take account of the potential impact of vaccination in reducing disease burden. The most current estimate of PCV impact on disease incidence is from a study in Sweden, which compared IPD incidence in the post-vaccine period (2013–2016) with the pre-vaccine period (2007–2009).^16^ This publication was selected because, in the absence of head-to-head studies comparing available PCVs, this Swedish study offered a unique opportunity to evaluate the combined impact of higher-valent PCVs in a uniform population. Across all serotypes, in children aged 0–4 years the estimated incidence rate ratio was 0.37 (95% confidence interval [CI] 0.28, 0.49),^16^ which represents a 63% reduction in IPD incidence after PCV introduction (i.e. post-PCV-10/13 vs pre-PCV-7). We therefore multiplied the expected annual case numbers estimated using the incidence rates published by O’Brien et al.^14^ by 0.37 to estimate expected annual case numbers adjusted for the impact of PCV introduction.

## Methods: calculating the proportion of cases captured by the surveillance program

The annual number of isolates reported by SIREVA for each country was divided by the annual estimated number of cases expected in that country (adjusted for vaccine impact as described in the previous section), to calculate the percent difference between reported isolates and estimated cases.

## Results

**Figure 2.**
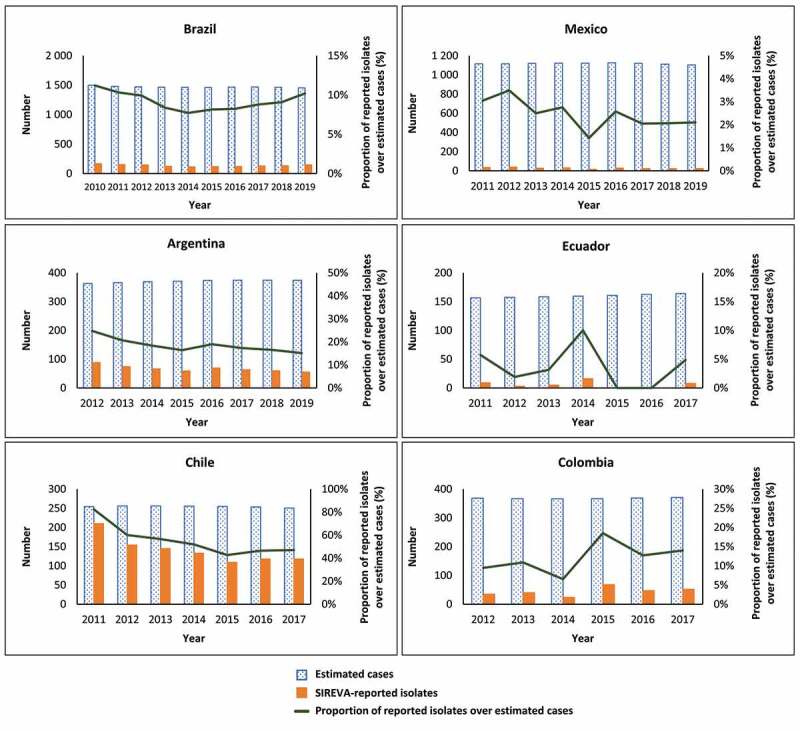
Country-specific SIREVA-reported isolates of pneumococcal meningitis and sepsis/bacteremia versus estimated expected number of cases adjusted for vaccine impact in children aged <5 years, and percent difference between SIREVA-reported isolates and estimated cases, from year of higher-valent PCV introduction to latest year for which data are available.

Figure 2 shows the number of isolates of pneumococcal meningitis and sepsis/bacteremia reported by SIREVA for each country and year and compares this with the estimated number of cases expected in each country and year. It is observed that the number of isolates reported by SIREVA was proportionally lower than the number of cases expected, across all six countries and all the years available. The number of SIREVA-reported isolates in relation to the estimated number of expected cases was highest in Chile and was particularly low in Mexico and Ecuador.

PCV, pneumococcal conjugate vaccineYears represent the post-vaccination years for higher-valent PCVs in all countries. Proportion of reported isolates over estimated cases represents the number of isolates reported by SIREVA out of the estimated number of cases based on regional incidence rates (% Reported = Reported Cases/Estimated Cases).

Figure 2 also shows the percent difference between SIREVA-reported isolates and estimated cases by country. SIREVA-reported isolates represented 25% or less of the estimated number of cases across all the countries except Chile, where the proportion ranged from 43% to 83% depending on the year. The country with the lowest proportion of reported isolates was Mexico, where the proportion of reported isolates was 1.4% to 3.5% of the estimated number of cases.

## Discussion

The purpose of this analysis was to explore potential differences between alternative epidemiological approaches to estimating the prevalence of disease, by considering the number of pneumococcal meningitis and sepsis/bacteremia isolates reported in SIREVA surveillance data in the context of the estimated number of cases derived from population incidence data. We needed to adjust for the potential impact of PCV vaccination as the disease incidence data preceded the introduction of PCV vaccination programs in Latin America, noting that the present analysis did not attempt to evaluate vaccine impact. Our results show that the number of isolates of pneumococcal meningitis and sepsis/bacteremia in children aged <5 years reported in the SIREVA surveillance data was consistently lower than the estimated number of expected cases, adjusted for the impact of PCV vaccination, across all six countries studied and all the years with available data. The proportion of SIREVA-reported isolates in relation to the estimated expected cases varied considerably between the countries. In Chile the percentage of recorded isolates ranged from 43% to 83%, whereas in all the other countries the percent difference was 25% or lower. In some countries the percent difference was lower, e.g. in Mexico it ranged from 1.4% to 3.5%. Reporting is not mandatory and therefore might vary between different countries and/or districts, and laboratory practices might differ between countries.

Our analysis has a number of limitations, arising from the limited data available. In three countries (Argentina, Brazil and Mexico) the SIREVA data are updated with clinical diagnosis information available in children aged <5 years up to 2019. However, in the other three countries (Chile, Colombia and Ecuador), up-to-date diagnostic data are not available. Clinical diagnosis information was reported only up to 2017, and reports after this date are not stratified by clinical diagnosis, reporting only overall samples by serotype and meningitis versus non-meningitis isolates. We could therefore not use data after 2017 from these countries, since our analysis required data on isolates of meningitis and sepsis/bacteremia. We would therefore be unable to identify any recent trends or changes in the pattern of SIREVA-reported isolates in relation to estimated expected cases in our analysis in these countries.

Another potential limitation is that the case numbers estimated from the O’Brien et al. incidence data^14^ are modeled estimates and therefore subject to some uncertainty. However, the data published by O´Brien et al. were obtained using distinct methods to estimate the pneumococcal disease burden. Data for meningitis, non-meningitis invasive disease came from a literature review of published papers from 1980 to 2005, and inclusion and exclusion criteria as well as a quality assessment were applied to the reported data. From a methodological perspective, the estimation model used by O´Brien et al.^14^ is quite robust.

Another limitation is that we used a regional incidence estimate from the whole Americas region^14^ to estimate the expected number of cases in the six countries. This could not take account of any country-specific differences in disease incidence. The regional incidence estimate was selected because the country-specific data available in Latin American countries come from SIREVA. Since SIREVA is a laboratory-based surveillance system, there is no estimation of the disease burden based on incidence rates as population denominators are not adequately defined. In addition, this estimate of regional incidence pre-dates the introduction of PCV in the Latin American region and therefore does not reflect the impact on disease incidence arising from PCV vaccination programs in the region. To adjust for the impact of vaccination we applied an adjustment factor to the expected number of cases. However, as data were not available for Latin America, the adjustment factor was derived from a study conducted in Sweden.^16^ Sweden offered a unique opportunity to evaluate the impact of PCVs in a uniform population, because the 21 counties in Sweden each decided which PCV would be used in their childhood vaccination program.

Passive laboratory-based national surveillance systems are likely to underestimate disease occurrence, and active case finding is recognized as likely to provide a more complete count of cases than passive reporting.^17^ Surveillance systems, particularly in developing countries, are often impaired by shortages of resources, lack of suitably qualified personnel, outdated or nonfunctioning laboratory equipment, poor roads and communications with consequent difficulties in transporting samples quickly to laboratories, poor co-ordination between multiple systems and lack of firm links to response measures, leading to many disease cases going unreported.^18^ A report published in India identified scarcity of standard culture media, improper sampling, prior use of antibiotics and conservation of cerebrospinal fluid and blood samples as challenges in laboratory assessment of pneumococcal isolates.^19^ Passive surveillance has been criticized for under-reporting, lack of representativeness in cases that are reported, lack of timeliness, sensitivity and/or specificity and incorrect diagnoses by physicians.^2^ It has been estimated that for common diseases, health departments may obtain reports on only 10–25% of disease cases occurring in the community.^2^ Our results for the recorded isolates in SIREVA as a percentage of the estimated number of cases are broadly consistent with this estimate in most of the countries in our analysis.

Passive surveillance can be used to measure disease trends over time, for example as a tool to assess vaccine impact, if hospital admission rates, health-seeking behavior and surveillance methods remain stable.^17^ The performance of a disease surveillance system may change over time due to several factors. For example, changes in case identification methods, laboratory practice, reporting behavior, and the catchment areas or catchment populations of sentinel hospitals could all influence the data recorded in a passive surveillance system.^17^ Laboratory-based surveillance systems have limitations as identification, reporting, and laboratory diagnosis of cases may not be uniform, differing between countries and between healthcare providers and hospitals within a country.^5^ Some factors may be affected by the act of introducing vaccination, for example if reporting increases as a result of increased attention to the disease following vaccine introduction.^17^ Vaccine transition periods may be difficult to interpret as a result of mixed schedules and indirect effects from previous vaccines, and other confounders such as disease seasonality, changes in vaccine effectiveness and/or uptake, and introduction of vaccination in specific population groups (e.g. pneumococcal vaccination in elderly people) may affect disease occurrence. Stronger communication between epidemiologists, clinicians and laboratories, in particular by making it compulsory to report IPD, could help to improve surveillance data and would facilitate future analysis.^5^ A study in Italy compared cases of vaccine-preventable invasive bacterial disease identified from hospital discharge records with cases notified to the national surveillance system between 2007 and 2016.^20^ Although the study found under-reporting to the surveillance system, the agreement between surveillance records and hospital data improved over time. For invasive meningococcal disease (IMD) the proportion of surveillance cases to hospitalizations recorded increased from 57% in 2007 to 92% in 2016, for invasive *Haemophilus influenzae* disease (IHD) the increase was from 35% to 87%, and for IPD the percentage increased from 45% in 2007 to 99% in 2014 and in 2015 and 2016 the number of cases reported to the surveillance system exceeded those in hospitalization data.^20^ During the study period, several initiatives were implemented to improve the notification rate, including defining a more sensitive laboratory diagnosis for confirmation, training and awareness-raising initiatives for relevant specialists, and recommendations for public health practitioners and health managers. In addition, in 2013 data from the national surveillance system were also integrated with data from regional infectious disease notification systems.^20^ The study concluded that efficient and reliable surveillance systems are fundamental for monitoring disease trends and outbreaks, and that efforts such as automated reporting and improved training could be implemented to reduce under-reporting and strengthen surveillance procedures.^20^

## Conclusion

SIREVA represents an important tool to monitor circulating pneumococcal serotypes and antibiotic resistance in Latin America. However, reporting is not mandatory and the analysis we have presented here suggests that only a limited proportion of pneumococcal meningitis and sepsis/bacteremia cases are captured by SIREVA surveillance. This under-reporting will limit precision and representativeness when monitoring disease incidence trends after vaccine introduction, and these limitations should be considered when assessing PCV impact in Latin America.

The percentage of cases reported to a passive surveillance system can be increased over time by strengthening surveillance procedures using approaches such as implementing automated reporting, improving training and raising awareness. Any future potential changes or improvements in reporting and laboratory procedures, while potentially improving the percentage of cases reported to SIREVA, will also limit the ability to interpret changes in pneumococcal disease epidemiology over time, including changes of vaccine serotypes and replacement.
